# Active compound chrysophanol of *Cassia tora* seeds suppresses heat-induced lipogenesis via inactivation of JNK/p38 MAPK signaling in human sebocytes

**DOI:** 10.1186/s12944-019-1072-x

**Published:** 2019-06-07

**Authors:** Hyuk Chul Kwon, Tae Yang Kim, Chun Mong Lee, Kwang Sik Lee, Kun Kook Lee

**Affiliations:** Songpa R&D Center, Coreana Cosmetics Co., Ltd, 6, Samgok 2-gil, Seonggeo-eup, Seobuk-gu, Cheonan-si, Chungcheongnam-do Republic of Korea

**Keywords:** Human sebocytes, Acne vulgaris, *Cassia tora* seed extract, Chrysophanol, MAPK pathways, Heat-shock

## Abstract

**Background:**

Heat induced by infrared (IR) radiation from sun exposure increases skin temperature and can lead to thermal and photo-aging. However, little is known about the relationship between heat induced by IR radiation and lipid biosynthesis in human sebocytes. This study investigated the expression of factors involved in lipid biosynthesis in human sebocytes exposed to heat. The effect of *Cassia tora* extract and chrysophanol, which is widely used as anti-inflammatory agent, on the heat shock effect in sebocytes was then examined.

**Methods:**

For the treatment, cells were maintained in culture medium without FBS (i.e., serum starved) for 6 h and then moved for 30 min to incubators at 37 °C (control), 41 °C, or 44 °C (heat shock). Culture media were replaced with fresh media without FBS. To investigate expression of gene and signaling pathway, we performed western blotting. Lipid levels were assessed by Nile red staining. The cytokine levels were measured by cytokine array and ELISA kit.

**Results:**

We found that peroxisome proliferator-activated receptor (PPAR)γ and fatty acid synthase (FAS) were upregulated and the c-Jun N-terminal kinase (JNK)/p38 signaling pathways were activated in human sebocytes following heat exposure. Treatment with *Cassia tora* seed extract and chrysophanol suppressed this up-regulation of PPARγ and FAS and also suppressed the increase in IL-1β levels.

**Conclusion:**

These findings provide evidence that IR radiation can stimulate sebum production; *Cassia tora* seed extract and chrysophanol can reverse lipid stimulated inflammatory mediation, and may therefore be useful for treating skin disorders such as acne vulgaris.

## Background

Human skin is composed of the epidermis, dermis, and a subcutaneous fat layer. Hair, hair follicles, and sebaceous glands form a pilosebaceous unit. Sebaceous glands produce and secrete sebum, which contains triglycerides, wax esters, squalene, cholesterol esters, cholesterol, and free fatty acids; excessive sebum production is associated with skin disorders such as acne [1, 2]. Sebaceous gland cell proliferation and sebum production are regulated by a complex system of hormones, as well as other factors such as genetics, environmental and metabolic conditions, stress, diet, and injury.

Heat from infrared (IR) radiation during sun exposure increases skin temperature. Infrared (IR) radiation is classified as IR-A (760–1400 nm), IR-B (1400–3000 nm), and IR-C (300 nm–1 mm). IR-A penetrates deep into subcutaneous tissues, whereas IR-B and -C are mostly absorbed by the epidermis [3]. When exposed to sunlight in the summer, the skin temperature increases by more than 40 °C. In addition, when exposed to a temperature of 45 °C or more for 1 h or more, skin tissue is damaged by protein denaturation in the skin.

A previous study reported that sebum production was increased by 10% for each 1 °C increase in skin temperature [4, 5]. However, the molecular basis for the relationship between heat and lipogenesis is not known in human sebocytes.

Of the three PPAR isoforms, activation of PPARγ is important for sebocyte differentiation and lipid production [6, 7]. PPARγ is regulated by its targer gene, Adipose differentiation-related protein (ADRP) and Angiopoietin-like 4 (ANGPTL4). Adipose differentiation-related protein (ADRP) is a major protein localized at the lipid droplets in most cell types and Angiopoietin-like 4 (ANGPTL4) is a secreted glycoprotein with established functions in lipid metabolism [8, 9].

Chrysophanol, the active substance of *Cassia tora,* is a member of the anthraquinone family. The results of previous pharmaceutical studies have shown that derivatives of anthraquinones exert a number of biological effects, including anticancer [10], hepatoprotective [11], and antimicrobial [12]. Also, chrysophanol decreased lipid accumulation and the expressions of adipogenesis factors including peroxisome proliferator-activated receptor gamma (PPARγ) and CCAAT/enhancer-binding protein alpha (C/EBPα) in 3 T3-L1 adipocytes [13]. These observations, together with the fact that acne is closely related to abnormal bacterial proliferation, inflammation, and lipogenesis, led us to hypothesize that chrysophanol may be beneficial in acne.

In this study, we investigated whether heat treatment induces lipid production in sebocytes, and whether this can be inhibited by *Cassia tora seed* extract (CTSE) and chrysophanol (CP).

## Methods

### Preparation of *Cassia tora* seed extract

*Dried Cassia tora* seed (cultivated in Youngcheon, Korea) were purchased from the local market. The dried sample was ground into powder and passed through a sieve (20 mesh). The seeds were ground into a fine powder and extracted with 70% ethanol in a stainless steel extraction tank for five days at room temperature. The ethanol mixture was filtered through a funnel and centrifuged (4 °C, 10,000 x *g*, 20 min), and this process was repeated three times. The precipitate was removed, and the ethanol extract was collected and concentrated using a rotary evaporator. Chrysophanol was purchased from Sigma-Aldrich (St. Louis, MO, USA).

### Cell culture

Human sebocytes were purchased from ZenBio (Research Triangle Park, NC, USA) and cultured in Sebomed medium (Biochrom^ag^, Berlin, Germany) supplemented with 10% fetal bovine serum (FBS; HyClone, Logan, UT, USA) and 5 ng ml^− 1^ recombinant human epidermal growth factor (Biochrom^ag^, Berlin, Germany). For the treatment, cells were maintained in culture medium without FBS (i.e., serum-starved) for 6 h and then moved for 30 min to incubators set at 37 °C (control), 41 °C, or 44 °C (heat shock). Culture media were replaced with fresh media without FBS and the cells were further incubated for 48 h [14].

### Assessment of cell viability

Human sebocytes (1 × 10^4^ cells per well) were seeded into 48-well plates. The next day, the cells were treated with various concentrations of CTSE (0–100 ppm) or CP (0–100 μM) and subsequently incubated for 24 h. Cytotoxicity was assessed by adding 5 mg/mL MTT reagent (3-(4,5-dimethylthiazol-2-yl)-2,5-diphenyltetrazolium bromide) to the cells for 4 h, after which the precipitated formazan was dissolved with dimethyl sulfoxide and the optical density (OD) was measured at 560 nm using a spectrophotometer.

### Western blot analysis

Cells were lysed in Pro-prep solution (Intron, Daejeon, Korea). Proteins in the lysates were resolved by sodium dodecyl sulfate-polyacrylamide gel electrophoresis and transferred to a nitrocellulose membrane that was then incubated with rabbit monoclonal antibodies against the following proteins: peroxisome proliferator-activated receptor (PPAR)γ, JNK and phosphorylated (p)-JNK, p38, and phosphorylated (p)-p38, Nuclear Factor κ B (NF-κB) and phosphorylated (p)-NF-κB, inhibitor kappa B-alpha (IκBα) and phosphorylated (p)-IκBα (Cell Signaling Technology, Danvers, MA, USA), fatty acid synthase (FAS), Angiopoietin-like 4 (ANGPTL4), Adipose differentiation-related protein (ADRP) (Santa Cruz Biotechnologies, Santa Cruz, CA, USA), and interleukin (IL)-1β (Abcam, Cambridge, UK). The blots were then incubated with peroxidase-conjugated secondary antibodies and visualized with a C-DiGit Chemiluminescent Western Blot Scanner (LI-COR Biosciences, Lincoln, NE, USA).

### Nile red staining

To detect sebaceous lipids, human sebocytes were seeded in 12-well culture plates at a density of 1 × 10^5^ cells per well and cultured overnight, after which they were treated every two days with CTSE or CP for a total of four days. For Nile Red staining, a stock solution of Nile Red (Sigma-Aldrich, St. Louis, MO, USA; 1 mg/mL in acetone) was diluted to a final concentration of 10 μg/mL in phosphate-buffered saline (PBS). Cells were fixed in 4% formaldehyde at room temperature for 10 min, stained with Nile Red solution for 15 min at 37 °C, and washed with PBS. Stained cells were visualized by fluorescence microscopy using 485-nm excitation and 565-nm emission filters. Lipids were quantitatively measured using Leica Application Suite (LAS) X software.

### IL-1β ELISA

To assess cytokine release, the IL-1β level was measured with an ELISA kit (R&D Systems, Minneapolis, MN, USA) (sensitivity < 250 and < 3.9 pg/mL) according to the manufacturer’s instructions.

### Cytokine array

The levels of 42 human cytokines were analyzed using the Human Cytokine Antibody Array (Abcam, Cambridge, UK). After heat shock, human sebocytes were treated with CP (0–100 μM) for 48 h, and then culture supernatants were analyzed for the presence of cytokines according to the array manufacturer’s instructions. Briefly, membranes were blocked for 30 min and then incubated with culture supernatant for 2 h at room temperature. Membranes were then washed with washing buffer and incubated overnight at 4 °C with a 1× biotin-conjugated antibody mix. After additional washes, streptavidin-conjugated peroxidase was added to the membranes followed by incubation for an additional 2 h. After thorough washing, the membranes were visualized with the C-DiGit Chemiluminescent Western Blot Scanner (LI-COR Biosciences).

### Statistical analysis

All experiments were repeated at least three times with different batches of cells. Data were evaluated statistically using Student’s t-test. Statistical significance was set at *P* < 0.05.

## Results

### Heat treatment stimulates lipid synthesis in human sebocytes

**Fig. 1:**
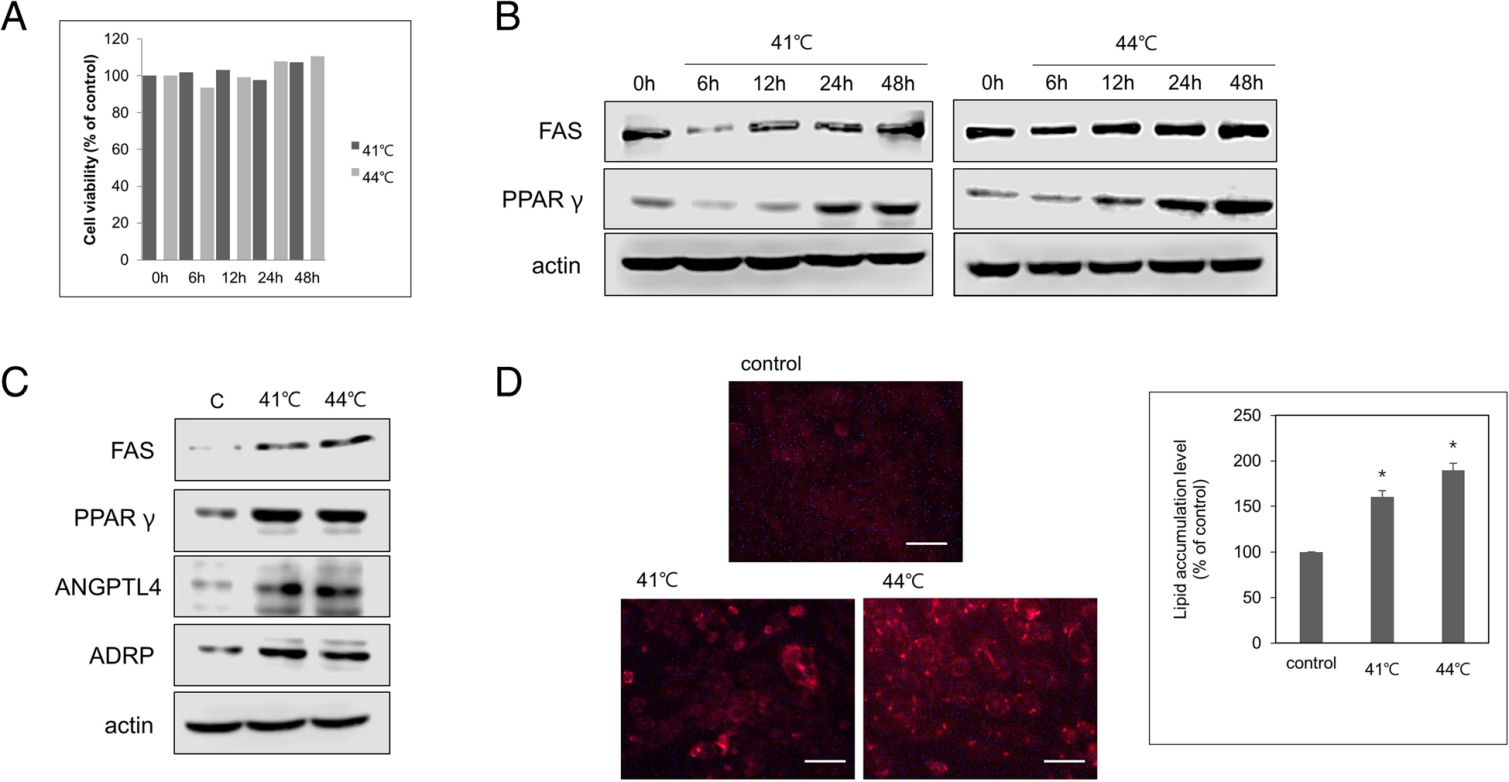
PPARγ and FAS are upregulated by heat in human sebocytes. Human sebocytes were serum-starved for 6 h and then incubated for 30 min at 41 °C or 44 °C. Fresh culture medium was added and the cells were cultured. **a** Cell viability was assessed by MTT assay. **b**, **c** PPARγ, FAS and target gene of PPARγ protein levels were analyzed by western blotting, with actin used for normalization. **d** Intracellular lipids in human sebocytes exposed to heat were assessed by Nile Red staining. Scale bar = 20 μm. Quantitation of lipid levels in human sebocytes exposed to heat, calculated as percentage of the value in cells incubated at 37 °C. Data represent the mean ± SE (*n* = 3). *P < 0.05, (Student’s t test)

We first examined the cell viability of human sebocytes after heat treatment, Human sebocytes were cultured at 41 °C or 44 °C for 30 min. Cell viability was assessed by MTT assay, and found that cell viability was unaffected in over time the cultured human sebocytes incubated at 41 °C or 44 °C for 30 min (Fig. 1a). Next, we examined whether PPARγ and FAS play a role in the increase in lipid production in human sebocytes caused by heat treatment.

Human sebocytes were cultured at 41 °C or 44 °C for 30 min and showed increased PPARγ and FAS levels over time (Fig. 1b). As shown in Fig. 1c, ADRP and ANGPTL4, which are known to modulate PPARγ, also increased at 41 °C or 44 °C. To investigate the effect of heat on lipid synthesis in human sebocytes, we examined lipid droplet formation using Nile Red staining and found that cells incubated at 44 °C showed higher lipid accumulation than those incubated at 41 °C (Fig. 1d). These results suggest that heat may induce lipogenesis in human sebocytes.

### CTSE and CP suppress heat-induced lipid synthesis

**Fig. 2:**
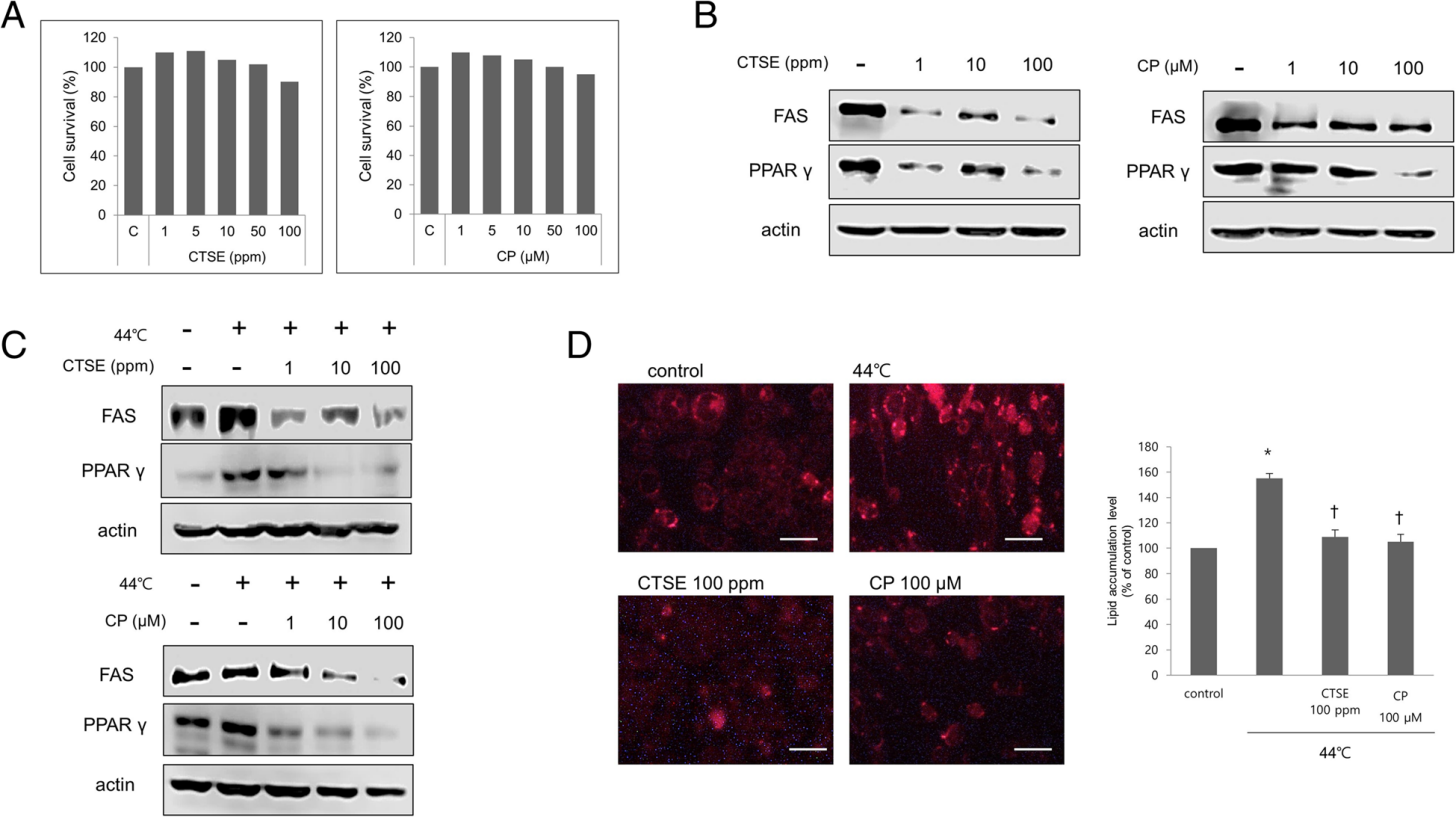
Inhibition of lipid synthesis by CTSE and CP in heat-treated sebocytes. Human sebocytes were serum-starved for 6 h and then incubated for 30 min at 44 °C. Fresh culture medium was added with various concentrations of CTSE (0–100 ppm) or CP (0–100 μM), followed by incubation for 48 h. All experiments included non-heat-shocked and untreated sebocytes as a control. **a** Cells were treated with various concentrations of CTSE (0–100 ppm) and CP (0–100 μM), and cell survival was analyzed using the MTT reduction assay. Data represent the mean ± SD of triplicate samples expressed as a percentage of the control. **b**, **c** Cell lysates were analyzed by western blotting for PPARγ and FAS with actin used for normalization. **d** Levels of intracellular lipids assessed by Nile Red staining. Scale bar = 20 μm. Lipid levels in heat-shocked and CTSE or CP-treated human sebocytes, calculated as a percentage of the value in cells incubated at 37 °C. Data represent the mean ± SE (*n* = 3). *P < 0.05 vs. cells incubated at 37 °C; ^†^P < 0.05, vs. cells incubated at 44 °C, (Student’s t test)

The ability of CTSE and CP to inhibit lipid production was also examined to inhibit lipid production induced by heat treatment at 44 °C, and found that cell viability was unaffected at concentrations up to 100 ppm and 100 μm, respectively, as determined using the MTT assay (Fig. 2a). we examined the effect of CTSE and CP tested on their own without heat exposure. As shown in Fig. 2b, both CTSE and CP suppressed expression of PPARγ and FAS in a concentration-dependent manner (Fig. 2b). Additionally, The upregulation of PPARγ and FAS upon heat treatment was reversed by CTSE and CP in a concentration-dependent manner (Fig. 2c). To examine the effect of CTSE and CP in greater detail, we examined lipid accumulation in sebocytes by Nile Red staining and found that CTSE and CP reduced lipid accumulation in heat-treated cells (Fig. 2d).

### Heat induces lipid synthesis via activation of JNK/p38 signaling, and this effect is suppressed by CP

**Fig. 3:**
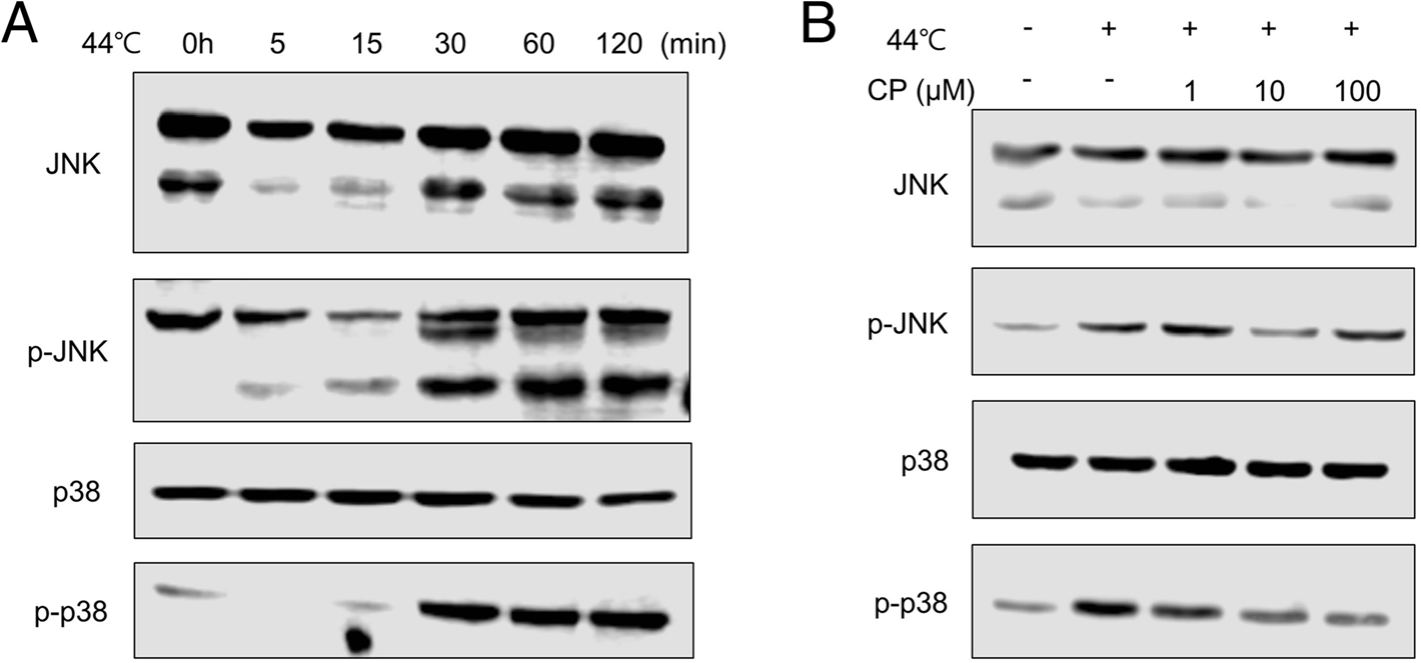
Heat induces lipid synthesis in human sebocytes via activation of JNK/p38 signaling. Human sebocytes were starved for 6 h and then incubated for 30 min at 44 °C. Fresh culture medium was added and incubated. **a** Whole cell lysates were prepared in a time-dependent manner and analyzed by western blotting for JNK and phospho-JNK, and p38 and phospho-p38. **b** Human sebocytes were incubated for 30 min at 44 °C and treated with various concentrations of CP (0–100 μM), followed by incubation for 48 h

To clarify the molecular mechanism by which CP suppresses lipid synthesis, we investigated the signaling pathway that was activated in human sebocytes exposed to heat and found that the levels of phospho-JNK and phospho-p38 increased in time dependent manner at 44 °C (Fig. 3a), and this activation was concentration-dependently abrogated by CP (Fig. 3b). Our results indicate that activation of JNK/p38 may be associated with heat-induced lipogenesis in human sebocytes.

### CP regulates inflammatory cytokine production in heat-treated human sebocytes

**Fig. 4:**
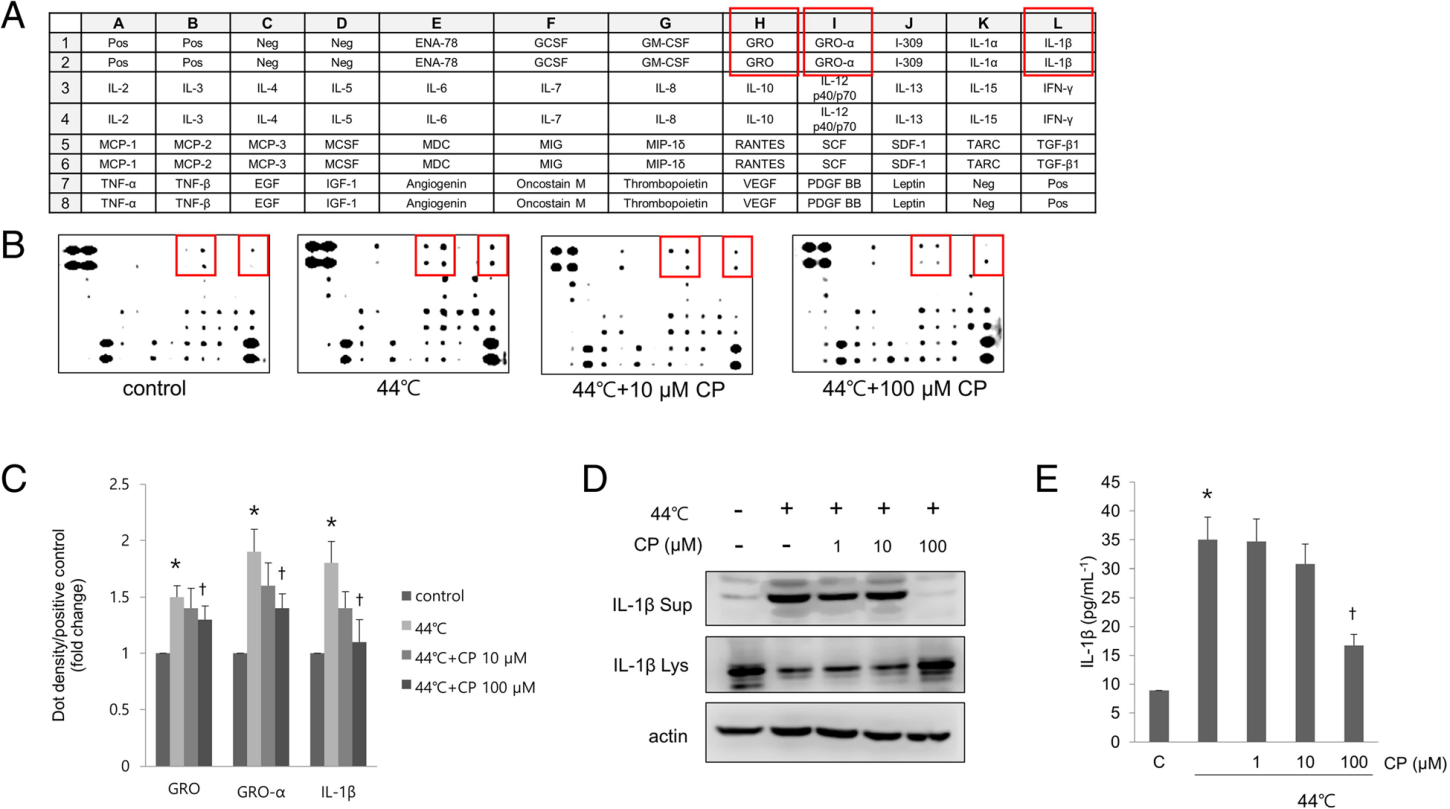
Reduction of pro-inflammatory cytokine levels by CP treatment of heat-shocked human sebocytes. Human sebocytes were serum-starved for 6 h and then incubated for 30 min at 44 °C. Fresh culture medium was added with various concentrations of CP (0–100 μM), followed by incubation for 48 h. **a** Cytokine levels were detected with an array that included antibodies against 42 different cytokines. Neg, negative control; Pos, positive control. **b** Three pro-inflammatory cytokines were upregulated by heat treatment (red squares); this effect was mitigated by treatment with CP. **c** Densitometry analysis of cytokine levels. **d** Pro-IL-1β protein levels in the whole cell extract and mature IL-1β protein level in the culture supernatant were determined by western blotting, with actin used for normalization of the whole cell lysate. **e** Mature IL-1β in the culture supernatant was detected by ELISA. Data represent the mean ± SEM (*n* = 3). *P < 0.05 vs. cells incubated at 37 °C; ^†^P < 0.05 vs. cells incubated at 44 °C, (Student’s t test)

To investigate the effects of CP on human sebocytes in greater detail, we examined the expression of various pro-inflammatory cytokines using a cytokine array (Fig. 4a). we observed that growth-regulated oncogene, growth-regulated oncogene-a, and IL-1β were upregulated by heat treatment, and that the levels were reduced by CP. IL-1β was already known to be upregulated in acne lesions (Fig. 4b, c) [15]. To confirm these results, we evaluated IL-1β expression by western blotting and ELISA. The lysates of heat-treated human sebocytes showed elevated IL-1β levels as compared to those of cells incubated at 37 °C; however, this increase was abolished by CP treatment, as determined by western blotting (Fig. 4d) and ELISA assay (Fig. 4e).

### Heat induces IL-1β–mediated NF-kB signaling in human sebocytes

**Fig. 5:**
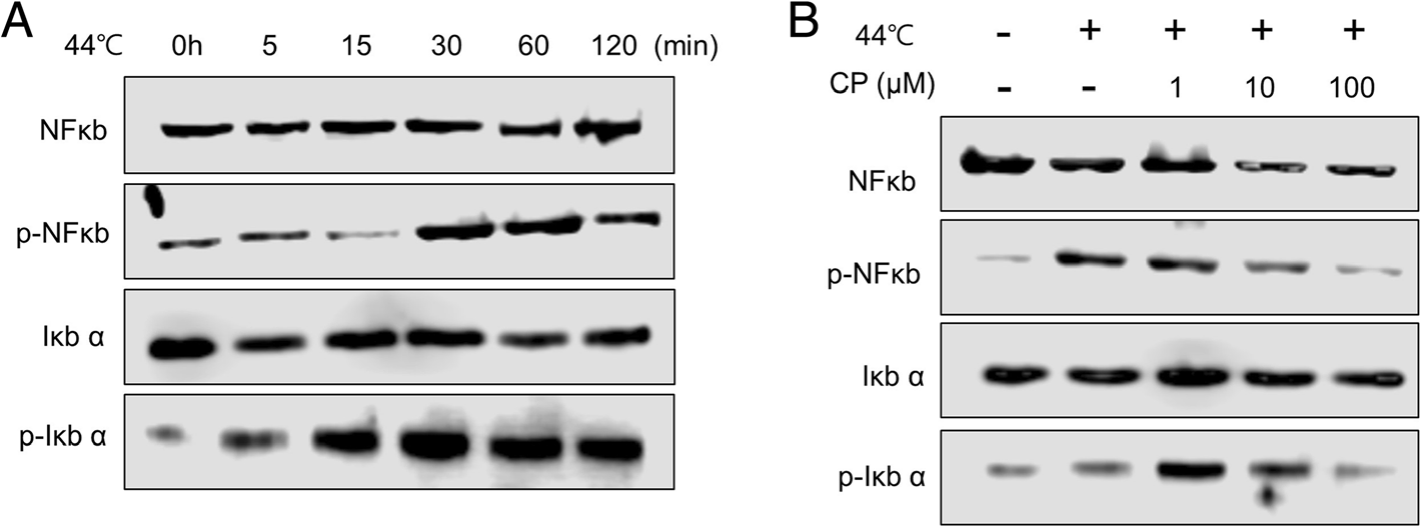
Expression of NF-kB signaling by heat treatment in human sebocytes. Human sebocytes were starved for 6 h and then incubated for 30 min at 44 °C. Fresh culture medium was added and incubated. **a** Whole cell lysates were prepared in a time-dependent manner and analyzed by western blotting for NFκB and phospho- NFκB, and IκB α and phospho- IκB α. **b** Human sebocytes were incubated for 30 min at 44 °C and treated with various concentrations of CP (0–100 μM), followed by incubation for 48 h

To determine the molecular mechanism of the effect of heat on IL-1β–mediated cytokine expression in human sebocytes, we investigated the the NF-kB and IkBα pathway that was activated in human sebocytes exposed to heat and found that the levels of phospho-NF-kB and phospho-IkBα increased in time dependent manner at 44 °C (Fig. 5a), and this activation was concentration-dependently decreased by CP (Fig. 5b).

## Discussion

Acne vulgaris is among the most common chronic skin diseases. The pathophysiology of acne is complex, involving abnormal keratinization of keratinocytes and proliferation of the causative bacterium *Propionibacterium acnes* resulting from perturbation of sebaceous gland function and the resultant increase in sebum production [16]. Androgen hormones also regulate sebaceous gland function by binding to nuclear androgen receptors [17].

It has previously been reported that sebum secretion is increased by ultraviolet (UV) radiation and that IL-1α, −1ra, − 6, and − 10 production is upregulated in acne patients [18]. It was also shown that IL-1β and − 8 expression was induced by UVB in cultured sebocytes [19], and that sebocyte proliferation and sebum production were markedly increased in cultured hamster sebocytes exposed to UVB radiation [15]. However, UVA radiation, visible light, and IR light did not alter pro-inflammatory cytokine expression [20], and another study found that near-IR radiation did not affect sebum secretion in hamster sebocytes [4].

In the present study, we found that PPARγ and FAS were upregulated, and lipid accumulation was increased, in human sebocytes after exposure to heat in a temperature-dependent manner. Moreover, ADRP and ANGPTL4, which are known to modulate PPARγ, also increased by heat treatment. They may be mediated by JNK and p38 phophorylation.

The JNK and p38 mitogen-activated protein kinase (MAPK) signaling pathways play an important role in basic cellular processes including proliferation, differentiation, survival, and migration [21, 22]. Also, JNK seems to mediate lipogenesis in several cell types, as presented by other studies. JNK is involved in KGF-induced lipogenesis in keratinocytes by increasing SREBP-1 mRNA expression and protein maturation [23], and hepatocytes also utilize JNK as a lipogenic signal molecule [24]. It has been reported that p38 is an adipogenic signal molecule and specific p38 inhibitors blocked adipogenesis and FAS expression [24]. Furthermore, p38 mediates lipogenesis in hepatocytes [25]. Here we found that JNK/p38 signaling was activated by heat in human sebocytes and that this effect was inhibited by CP treatment. These results indicate that heat stimulates lipogenesis and sebum production, and that this effect can be suppressed by CTSE and CP.

In a previous study, Stimulation of sebocytes by *P. acnes* increased the expression of the pro-inflammatory cytokines IL-1α, −1β, − 6, and − 8, which have a key role in acne pathogenesis [26, 27]; however, there is no evidence that a similar effect in sebocytes leads to acne. We also showed that IL-1β, a pro-inflammatory cytokine, was upregulated by heat in human sebocytes, and that CP suppressed this effect. IL-1β mediated NFkB activation proceeds through a pathway involving phosphorylation and subsequent degradation of the NFkB inhibitor (IkB α), resulting in the cytoplasmic release and nuclear translocation of NFkB [28]. In this study, the expressions of phosphorylated IkB α and NFkB in human sebocytes were upregulated in response to heat stimulation. However, CP drastically downregulated the increased expression of phosphorylated IkB α and NFkB by heat. Therefore, CP exerted an anti-inflammatory effect through modulating the NFkB pathway.

In conclusion, we have demonstrated that exposure to IR radiation induced lipogenesis and sebum production via activation of JNK/p38 MAPK signaling in human sebocytes, and that this effect that was mitigated by CP. These results suggest that an increase in skin temperature caused by excessive sun exposure can promote skin disorders such as acne vulgaris and that CP is a candidate agent for the treatment of this condition.

## Data Availability

All data generated or analysed are included in this paper.

## Abbreviations

ADRP: Adipose differentiation-related protein (ADRP)
ANGPTL4: Angiopoietin-like 4
CP: Chrysophanol
CTSE: *Cassia tora* seed extract
FAS: Fatty acid synthase
FBS: Fetal bovine serum
IkBα: Inhibitor kappa B-alpha (IκB α)
IL-1: Interleukin (IL)-1β
IR: Infra-red
JNK: c-Jun N-terminal kinase
MAPK: Mitogen-activated protein kinase
NFkB: Nuclear Factor κ B (NFκB)
PPARγ: Peroxisome proliferator-activated receptor γ
